# The Role of CGRPin Nociception?

**DOI:** 10.1100/tsw.2001.421

**Published:** 2001-12-12

**Authors:** R. G. Hill

**Affiliations:** Neuroscience Research Centre, Merck, Sharp and Dohme (UK), Terlings Park, Harlow, CM20 2QR, UK

## Abstract

The failure of NK1 receptor antagonists to show analgesic activity in clinical trials in spite of abundant preclinical evidence for a role of this neuropeptide in nociception, makes it somewhat dangerous to speculate on the nociceptive role of other neuropeptides, especially with respect to therapeutic utility of receptor antagonists! However, CGRP is the primary afferent peptide with the strongest evidence of a role in pain perception. It is found in a greater proportion of sensory neurones than other peptides and is a constituent of A[delta ] as well as C-fibres. Inflammation of peripheral tissues upregulates production of CGRP in sensory ganglia, coincident with the development of hyperalgesia, and CGRP knockout mice have attenuated hyperalgesic responses. CGRP is released into the dorsal horn of the spinal cord (DHSC) by noxious peripheral stimuli and excites nociceptive DHSC neurones on local application. The peptide antagonist CGRP8-37 blocks the response to exogenous CGRP and can reduce the response of DHSC neurones to noxious peripheral stimuli. CGRP8-37 has also been shown to have behavioural antinociceptive properties when given intrathecally. Conversely, injection of CGRP itself to the PAG or n. accumbens has been reported to have antinociceptive effects that are reversed by CGRP8-37. With the advent of potent non-peptide antagonists such as BIBN4096BS we should soon be able to determine whether systemic blockade of all CGRP receptors produces antinociception without limiting side effects.

